# The systemic neoliberal colonisation of higher education: a critical analysis of the obliteration of academic practice

**DOI:** 10.1007/s13384-023-00613-z

**Published:** 2023-03-01

**Authors:** Christine Morley

**Affiliations:** School of Public Health and Social Work, Queensland University of Technology, Brisbane, QLD Australia

**Keywords:** Neoliberal managerialism, Neoliberal colonisation, Higher education, Narrative ethnography, Critical analysis

## Abstract

Within the neoliberal university, scholarship, education, students, academic staff, and practices are subordinated to managerial imperatives. University educators are denigrated and displaced by colonising neoliberal practices that systemically invalidate and invisibilise academic work. The present article provides an example of this by critically analysing the corrosive and Orwellian operations of neoliberal managerialism in higher education through the prism of my own experience of applying for ‘recognition of leadership’ in relation to teaching. I use a narrative ethnographic approach to generate new insights into the obliteration of academic practice in contemporary university contexts and to produce a counter-hegemonic discourse for understanding these processes. Following Habermas *inter alia*, it is argued that without radical reform, the uncoupling of the ethical and substantive dimensions of the (educational) lifeworld from systemic (neoliberal managerial) strategising will leave higher education in a state of paralysis. The analysis highlights the urgent need for resistance and provides a critical framework for academics to recognise and contest similar colonising processes occurring in their own experiences and contexts.

## Introduction

It is now well established that within contemporary university contexts, neoliberal managerialism diminishes scholarship, education, students, and academic staff (see, e.g., Hil et al., 2022; MacDonald-Vemic & Portelli, 2020; Morley et al., 2017; Sims, 2020) by denigrating academic practices and reducing their worth to market calculations that seek to manipulate and exploit profit (Blackmore, 2014; Connell, 2013; Giroux, 2011, 2015). Neoliberalism is the discourse that justifies global capitalism as the dominant socio-economic system and seeks to extend capitalism to all spheres of life (Morley et al., 2019a). It involves concentrating wealth upwards, cutbacks to social provision, privatization, devolution of costs, and the imposition of individual responsibility for entitlements that used to be state obligations (Abramovitz & Zelnick, 2021). Managerialism is the set of practices that enact neoliberal discourses within workplaces and organisational contexts in ways that are profoundly undemocratic (Giroux, 2011); instead emphasising increased productivity, efficiency, and standardisation (Abramovitz & Zelnick, 2021). In the context of higher education, neoliberal managerialism deprofessionalises academic practices by conflating them with managerial strategies and techniques that require no disciplinary expertise; just indoctrination into neoliberal goals and managerial practices.

This article provides an example of this unsustainable situation by critically analysing dominant discourses within contemporary Australian university cultures. Inspired by Howie (2020), who critically reflects on his experience as a principal within a secondary school to critique and engender ‘grassroots’ contestation among educational leaders against the ‘bureaucratic managerialism in discourses of educational leadership’, I similarly reflect on my experience of attaining ‘recognition’ as an educational leader from a prestigious international institute, with the hope of cultivating resistance among academics (p. 279). While one could imagine successful recognition would value educational contributions; conversely, this critical enquiry highlights the invisibilising processes that obliterate academic practices.

## The proliferation of schemes that purport to ‘recognise’ academic practice

Recently, I was instructed by my manager to submit an application that, if accepted, would result in ‘international recognition’ of my ‘educational leadership’. As a passionate educator with considerable experience in applying critical pedagogies within social work education, I felt excited about the opportunities this process might afford for reflecting on and deepening my teaching and curricula development practices.

In addition, university educators are increasingly expected to have acquired this kind of recognition, which is tantamount to formally qualifying as a university teacher. As one academic observes in commenting on a similar kind of accreditation scheme: ‘we have seen sharp rises in the proportion of staff in the sector who are qualified to teach, so that it is now normal (if not quite prevalent) for early career academics to apply for jobs with a [award from the scheme] next to their PhD’ (Carter, 2017, p. 1). For academics that value education, Carter (2017) asserts that documenting educational achievements ‘is a necessary process if teaching is to gain parity with research’ (p. 4). This is important as within Australian universities (and within many other Anglophone countries), research, or more specifically research funding, and the commercial patents that this might generate, have become disproportionately valued, while teaching has been degraded and is often used as punishment for staff who are not seen as research-active (Hil, 2012; Morley et al., 2017).

Similar to my experience of preparing an analogous application, Carter, an academic who embarked on a similar process, described his experience as ‘time-consuming’, ‘horrendously self-indulgent’, and strange (Carter, 2017, p. 3). As Carter (2017) states:

The oddness exists at several levels. In the first place the style of writing is odd: you need to combine the unabashed self-praise of a job application with moments of raw self-reflection; secondly, this all needs to be a [*sic*] set in its proper scholarly context, which (unless your subject is Higher Education) is not likely to be your home turf; and, thirdly, the whole thing needs to be mapped and cross-referenced against the [scheme’s] values, skills and knowledge. (p. 46)

The application also requires three written referees who are also required to speak directly to the scheme’s criteria, essentially forcing them to engage with and be indoctrinated into the scheme as well. At the highest level, Carter (2017) further notes that the program:

Includes heroic expectations that a colleague has variously championed the [scheme’s criteria] and put the conditions in place for colleagues to follow it [and] are also expected to have had an impact on the student experience and on student learning at an institutional, sector-wide or even international level. (p. 2)

Obviously, within this criterion lies certain unstated and questionable assumptions about what is valuable about educational leadership.

Unlike my experience, this academic judged the scheme for which he applied as providing ‘professional recognition of underlying expertise... because it allows the individual academic to recognise his or her [*sic*] own expertise and achievements’ (Carter, 2017, p. 3). Here is where our experiences differ. Despite being ‘successful’, this reflection articulates how my expertise was systematically devalued, while my achievements and evidence of their impact were ultimately expunged from my application, to instead make way for a superficial description of management techniques designed to manipulate and coerce others for strategic ends.

## Method

The reflective process adopted in this inquiry is a narrative form of autoethnography (Ellis et al., 2011). Ellis et al. (2011) describe this approach as analysis of ‘texts presented in the form of stories that incorporate the ethnographer’s experiences into the ethnographic descriptions and analysis of others’ (p. 278). The texts that were analysed in this study compare my original submission with the final, ‘successful’ version, while taking account of feedback that I received along the way. Similar to a reflexive, deconstruction process within critical reflection (Fook, 2016), this comparison enabled the surfacing of dominant assumptions about what was valued versus what was discarded; what was silenced versus what was privileged; and what was distorted—and how and why. The ‘others’ in my narrative are the neoliberal administrative managers who acted as coaches and reviewers of my application and represent hegemonic discourse. Ellis et al. (2011) further state: ‘the emphasis is on the ethnographic study of others, which is accomplished partly by attending to encounters between the narrator and members of the groups being studied’ (p. 278). As they point out, ‘the narrative often intersects with analyses of patterns and processes’ (Ellis et al., 2011, p. 278). Themes and patterns in this study were identified by drawing upon insights from Orwell (1949) and subsequently theorised using Habermas’ (1984) concepts of system and lifeworld. While not the dominant research paradigm, reclaiming autoethnography and the critical analysis of experience as methodological strategies have something valuable to offer to educational research (Howie, 2020). In this case, the inquiry generated new insights into the insidious and destructive colonising neoliberal technologies operating in higher education and contributes to the unmasking of these technologies, which provides a critical analytic framework for academics to ‘engag[e] in counter-conduct and resistance to the practices of neoliberal governmentality’ (Selkrig et al., 2021, p. 733).

## My initial draft and case for recognition

While beyond the scope of this paper to explore the multiple (8 × 7000 word) iterations of the submission, there were certain examples of my academic practice that I thought were worthy of inclusion: my pedagogical approach; key teaching-related leadership roles; student and peer feedback on my teaching; institutional and national awards for teaching; and key publications that contribute to the scholarship of learning and teaching. While the application included other examples, I considered these the most compelling and have therefore emphasised them in this account.

I started the writing process with great enthusiasm. I was assigned one coach who moved on from the university, and then was allocated another. Both were well-meaning, supportive, and generous with their time. I was keen to learn from them, and I would not have been ‘successful’ in this endeavour without their counsel. Both were in senior administrator/management roles and had no direct teaching experience and little (if any) experience in leading curricula and/or engaging in research and publication. However, both had received international recognition at the highest level for their strategic leadership in relation to teaching and learning from this scheme and were now obviously involved in the mentoring of others.

I began the application by outlining my teaching philosophy and its purpose—that is, critical pedagogy to facilitate transformative learning and catalyse effective action for social justice and democracy (Brookfield, 2017; Freire, 1972; Giroux, 2011; Morley et al., 2020). I talked about the necessity of critical analysis and critical reflection (Morley et al., 2019a, 2019b, 2020). In practical terms, I talked about embodying critical theory and about the need to decentre emphasis from myself as the educator; create non-hierarchical relationships between myself and students and colleagues; engage in participatory, dialogical, collaborative approaches to learning; and model anti-oppressive practices (Giroux, 2011; Morley & Ablett, 2020). At every point, I sought to link critical theory to every facet of my pedagogic practice. Rather than simply make claims, I knew I had to demonstrate the impact of my work with evidence—as I had been required to do previously when nominating for teaching awards and applying for promotion or new positions.

I aimed to clarify the practical impacts of my pedagogy by providing examples of how critical education catalyses conscientisation and transformative learning, resulting in students and staff working together as citizen activists to build a democratic society (Giroux, 2011; Morley, 2019; Morley & Ablett, 2020). To illustrate further, I included two photos with my colleagues and our students engaged in social action.

Following this, I outlined my ‘strategic leadership’ to enhance critical curricula through the development and leadership of two nationally accredited schools of social work. To underline the impact of these programs, I included extracts from unsolicited testimonials I had received from former students; sometimes years after they had graduated, who described the often life-changing impacts of their education and the implications for their professional practice. I also referred to commendations made about my leadership by the external professional accreditation body.

In seeking to demonstrate my impact internationally, as the application required, I chose to talk about two of my books that I believe make different contributions to teaching. This first example discussed my lead authorship of an introductory textbook, (Morley et al., 2014, 2019a) co-authored by (Selma Mafcarlane and Phillip Ablett). I described the purpose of this book as aiming to shape the social work discipline along more emancipatory lines, by offering educators a counterhegemonic alternative to mainstream approaches.

The second example I referred to was my lead editorship of an international handbook about critical pedagogies (Morley et al., 2020). I argued that this work traversed new territory by expanding the application and utility of critical theories in social work education. Consistent with the overarching vision that applicants must articulate, I suggested that the book is part of a strategic agenda for change by providing a robust and much needed alternative paradigm to the technique-driven ‘conservative revolution’ (Garrett, 2010, p. 340) currently being fostered by neoliberalism. As with the textbook, I included published reviews and testimonials (see, e.g., Simpson, 2020) and an image of the cover. Incidentally, I had to fight to keep both books in the application as I was initially told that ‘one was probably enough’. In subsequent iterations, I was advised that greater differentiation was necessary between the books. This surprised me. I emphasised that one is an authored, introductory textbook designed largely for students; the other is an edited international handbook designed to enhance academics and educators’ teaching practices.

## Management ‘Newspeak’ and the processes of colonisation

Initial feedback received from my coach advised that while my pedagogic approach ‘sounded lovely’, it was ‘far too theoretical’ and should not be the focus of this application. I was informed that I had ‘lost my voice’ beneath my discussion of the pedagogy and needed to change all ‘passive voice’ to active. I couldn’t really understand this because although I felt revolted by the self-congratulatory, egotistical, and narcissistic tone that all academics are forced to adopt in talking about our work in the neoliberal university, I was also cognisant with the expected conventions and tried to conform. As the months in which I was ‘coached’ to work on this application unfolded, however, I discovered there is a whole new level of management ‘Newspeak’ that would completely obliterate my own voice (Orwell, 1949, p. 2).

In George Orwell’s *1984* commentary on the dystopian project to establish a totalitarian regime, he discusses the fabrication of a new language (i.e., Newspeak). In Bentley’s (2017) terms, Newspeak ‘involves the simplification and purification of the English language to the extent that it functions purely as a means of maintaining… power and control’ (para.7). As Orwell (1949) explains: ‘the whole aim of Newspeak is to narrow the range of thought’ (p. 29). Within the context of the neoliberal university, managerial Newspeak seeks to erase academic thought, freedom, and practices by ensuring that they are only talked about in terms of management and neoliberal discourses, which then begins to pervert their meaning and occlude their existence. This explains, for example, the increasing emphasis on curators or ‘learning design[ers]’ instead of teachers or educators (Li et al., 2016, p. 216).

Orwell (1949) observes that language has the capacity to corrupt thought and distort meaning and another strategy he outlines in *1984,* through the manipulation and corruption of language, is ‘doublespeak.’ Doublespeak is a discursive device to obscure meaning to the point where words actually become their opposites. So, the ‘Ministry of Love’ is actually where The Party enforces loyalty through repressive tactics like systematic brainwashing and through the extraction of confessions using violence and torture, for example. Or where the ‘Ministry of Plenty’ is actually responsible for the rationing of food and other resources.

In applying this concept of ‘doublespeak’ to theorise my experience, I learned that this application, ostensibly to recognise the highest level of contribution to learning and teaching, in reality, had nothing to do with education and everything to do with management. As panel feedback on my application stated: ‘across the application in general, your focus tends towards the specifics of teaching and learning practices, as well as your pedagogic research and scholarly outputs... however a more detailed analysis *of where and how you have led others, and strategically driven agendas* should be the focus of this application’ [emphasis added].

I was also advised that the description of my work moved on too quickly to evidencing its impact, (which was a problem), and that I should instead focus on *how* I did what I did. To dismiss the evidence about the impacts of work as not demonstrating leadership ensures that this type of recognition is not accessible for most academics, even senior ones with clear national and international track records in advancing education. Essentially, despite explicit reference to ‘teaching’, leadership according to this scheme can only be recognised if enacted by senior executives within the university that implement institution-wide policy. This was confirmed when I was told that demonstrating my strategic leadership was going to be ‘difficult to achieve’ for ‘front-facing academic staff’ like myself because, after all, we [academics] are ‘not in strategic roles’, even at professorial level, which is clearly not regarded as institutionally significant.

Having uncovered the ruse, I wanted to opt-out. I could identify with the sentiments expressed by one participant from Richard Hil’s (2012) study who commented on working in the neoliberal university: ‘there is little respect for us [academics] as professionals. Having to confront this sort of bureaucratic culture is so dispiriting. I feel totally disillusioned. My spirit has gone’ (p. 115). I explained my error in agreeing to embark on this process to my boss, but having already invested so much time and energy in the process, she instructed me to proceed.

So, I started to treat the requirement to engage in ‘doublespeak’ process as a sport—asking myself what is the most ridiculous, ruthlessly ambitious, economically-motivated, self-centred statement that I could articulate that might align with what they imagine educational leadership to be? I was aware of the work of neoliberalism on myself as we are all compelled to:

Translate [our] activities into financial terms, to seek to maximize productivity… to cut out waste, to restructure activities that [are] not cost-effective, to choose between priorities in terms of their relative costs and benefits, to become more or less like a financial manager of [our] own professional activities. (Macias, 2015, p. 255)

I felt acutely aware of ‘the micro-spaces of everyday, ordinary neoliberalism’ that were being imposed (MacDonald-Vemic & Portelli, 2020, p. 298). We think through language, and so when one’s own use of language and meaning is corrupted by dominant discourse, neoliberal ideology becomes reinscribed (MacDonald-Vemic & Portelli, 2020). At the same time, however, I was morbidly curious to learn more about the insidious processes of colonisation and the systematic redefinition of what constitutes educational leadership in a way that invalidates and invisibilises academic work. This, I believed, warranted further exploration. As Blackmore (2014) explains:

Criticality requires negotiating tensions between the particular/universal, working with and working over dominant orthodoxies while providing alternative ways of doing and seeing the world, about working within and on the rules of the game… Knowing the dominant does not necessarily mean adhering to its position, but it is necessary to playing the game. (p. 515)

## Revisions and the subordination of academic work to management techniques

As a critical scholar, I am accustomed to resistance from hegemonic power and mainstream proponents. The stripping back of critical theory to emphasise the technical aspects of teaching felt in Hil’s (2012) words: ‘antithetical to intellectual practice’ (p. 108). It moves teaching away from the realm of artistry, knowledge co-creation for transformative learning, and connection with others, to the domain of standardising procedures that seek to implement a ‘pre-determined approach to learning … directly linked to "learning objectives" … [that]… act as a kind of pedagogical straight jacket’ (Hil, 2012, p. 114; see also, Howie, 2020). As stated elsewhere:

The tyrannical influence of learning objectives has been referred to by some academics as the ‘death of teaching’; burdensome administration reducing creativity and spontaneity, controlling pedagogic practices, and excluding and/or invisibilising everything that does not conform to a template. (Morley et al., 2017, p.29)

However, this process was somewhat different. The feedback received indicated less of an ideological objection to critical social work, critical pedagogy, or even activism, provided that I removed the evidence and instead privileged process. And by *process*, I mean the meaningless minutiae or the ‘nitty–gritty’ of my work with colleagues; the details of which are so banal that I could scarcely imagine warranted mentioning. As the formal feedback stated: ‘you … spend relatively little time reflecting on your leadership journey… eas[e] back on the impact quotes and integrat[e] more reflection into the section. What did you do to lead others and how did you do it? What did you learn about yourself as a leader? What were your key challenges?’ To demonstrate my ‘strategic leadership’ my coach translated that I needed to talk about the challenges (even if there weren’t any), describe how I anticipated these, and strategically managed them.

In relation to the textbook, the reviewers/assessors were more concerned with how the publication came about. So, I included information about how the publisher approached me to write the text. They were not interested in what the book was about, nor the purpose that it serves, or how (or whether) it is used. Intriguingly, they were more interested in how I managed my co-authors to produce the book. The truth of the matter is that one of my co-authors has been one of my closest friends for more than two decades, and the other is a long-time best friend and colleague (and my husband). Working with Phillip (Ablett) and Selma (Macfarlane) was fun—spirited conversations and debate, laughter, encouragement, probably moments of panic as deadlines loomed, and support for each other, but certainly not a compelling case for educational leadership. It’s what we managed to achieve together that was remarkable (and, in my view, important for this application). However, I was coached to ‘find my voice’ and instructed to instead articulate a management process that ended up in the final application being expressed as:

I strategically adopted a collaborative approach to leadership where I fostered a culture of autonomy in which my co-authors could each contribute from their respective areas of strength and expertise… To ensure my co-authors did not feel overwhelmed, I endeavoured to be accessible and available and devised manageable tasks, through for example allocating topics we each needed to cover, based on our various strengths and interests… Another way I led my co-authors was to hold them accountable to deadlines to which we had collectively agreed.

It felt entirely foreign and alienating to talk about my organic and productive working relationships with my co-authors, with whom I have close, personal relationships, in such an instrumental, controlling, and contrived way. As Orwell (1949) suggests, the most successful way to annihilate people is to repudiate and erase their own understanding of their history. I felt this was occurring as I moved deeper into the neoliberal vortex of this application. And, while informally, perhaps even accidentally, I did lead this textbook project to a successful conclusion, the point is that the generic description of the management practice that was included in the final submission could have been narrated by anyone (or perhaps more pertinently, any manager)—and would not have necessarily produced the kind of book that we did with the impacts that it achieved—all of which were rendered invisible in this neoliberal reconstruction of ‘leadership’. This process perverts the fundamental nature value of academic knowledge and practice—’no longer treated as intrinsically valuable in its own right, but as a commodity that should be "leveraged" for profit’ (Fraser & Taylor, 2016, p. 12; see also, Berg et al., 2016; Morley et al., 2017; Sims, 2020). Imposing judgement about what is deemed important and what is rendered irrelevant, denies academic expertise and leadership, while concurrently reducing academic practices to robotic-like, decontextualised techniques; thereby deskilling academic practices and shunning academic outputs (Morley et al., 2019b).

And at the same time, I was being told to articulate how I ‘managed’ my co-authors in true neoliberalism fashion. I was simultaneously instructed to remove all references to ‘we’, and only refer to ‘I’. So, my collaborators were useful subjects to mention only insofar as to how I managed them to produce outputs, yet irrelevant in terms of their contributions to what we actually achieved. Giroux (2011) explains how neoliberalism produces ‘a ruthless competitiveness, an almost rabid individualism, and a notion of agency largely constructed within a market-driven rationality and economic growth’ (p. 9). Selkrig et al. (2021) further explain that neoliberalism ‘transforms relationships in universities into transactions that obscure the realm of emotions’ (p. 723). Implicitly, relationships are only valued when they are self-serving (Giroux, 2011; Morley et al., 2017). Rarely do we achieve anything without our collaborators, so to talk about ‘I’ instead of ‘we’ felt highly problematic, detestable in terms of values, and dishonest for a range of reasons. This process raises questions about the type of leadership that is being recognised in universities and the intent of this process. Garrett (2010) argues that neoliberalism penetrates the psyche of everything we do so deeply, that it embeds a new form of ‘common sense’, perhaps even changing not only how we work and organise society, but the nature of our souls (p. 343).

In terms of the textbook, my coach was also much more interested in the fact that we produced a companion website to accompany the second edition. So, I included information about this in the application, but again, this is such a minor aspect of authoring a book and completely misses the point about its purpose; not even the icing on the cake, let alone the cake—it so was unremarkable. The examples I was being instructed to emphasise were levelling academic expertise in a way that positions ethical discourse as window dressing. Essentially, the ‘what’ that we achieved didn’t matter, as long as we continued to reinforce the central messages of neoliberalism—the idea that leaders ‘should construct and produce self—enterprising individuals solely interested in enhancing their human capital’ (Shahjahan, 2014, p. 221, as cited in Fraser & Taylor, 2016, p. 5) by producing strategic outcomes for profit, and winning, regardless of moral terrain.

When discussing our handbook, my ‘much stronger and improved’ application was perhaps even more absurd and diminishing. No longer worth mentioning for its internationally recognised scholarly contributions to learning and teaching, it seemed to hold more ‘strategic value’ in the application as a ‘training manual’ for other academics or as an example of my ‘integrated approach’ to academic practice across teaching, research, and scholarship. Hence it was redesigned in the final application as a mechanism to ‘strategically support the teaching capabilities of social work educators to develop and deliver critical social work curricula’.

My emphasis on scholarly outputs, particularly books, was viewed as problematic at this point, and so it was decided it would be more ‘strategic’ to emphasise how I worked with my co-editors and contributing authors to develop a network of scholars to ‘promote the integration of the academic practices of others’. I should note that this aspect of the handbook project was achieved merely by sending a few emails, and again, it completely neglects the significance, purpose and value of this work, essentially reducing it all to a form of instrumental rationality (Weber, 1968). This resonates with Giroux’s (2015) characterisation of universities becoming ‘intellectual dead zones’ (pp. 122–123) and Orwell’s notion of being ‘taken over by a totalitarian regime in which the state [or in the case of universities, senior administrative managers] exert absolute power over its citizens’ [i.e., academics and students] (Bentley, 2017, para. 2).

My coach and the panel reviewers continued this (now familiar) pattern in relation to feedback about my leadership of academic programs. They were not interested in the fact that I had set up entire academic programs, or secured multiple national accreditations, or concerned about the nature of the programs, or what they contribute to student learning, or indeed to communities through graduates’ professional practice. None of these things were considered relevant to leadership in relation to teaching and learning. Equally, the university-level and national teaching awards did not merit a mention, nor did the independent evidence gathered from students, graduates, and accreditation bodies. All narratives about impact were removed from the application—just as published reviews and testimonials were deleted about the impact of the books (incidentally, the photos and images of book covers also had to be erased as I was informed that the application could only consist of text).

Instead, the review panel wanted to know *how* I had led my colleagues when establishing these programs, rather than about the development of curriculum, other relevant course matters, or the purposes that they serve. As the panel stated: ‘what would strengthen this section is a stronger articulation of your practice. That is, try and focus more on the process of leading. How did you work with others in the development of these new courses? How did the principle of theory to practice influence you as a leader?’ I recall my struggle to explain theory to practice, when my application was judged as too theoretical, my practice viewed as irrelevant, and any examples of substance had been removed.

They also questioned my role as a leader, indicating that I had described being part of a ‘thriving community of scholars’ (which I am), ‘rather than a leader of these scholars’ (which I also am). Apparently, in management doublespeak, there is no room for one to occupy the subject positions of both colleague and leader. This irritated me no end as I was pained to articulate ‘My strategic priority’ [as being] ‘to leverage my leadership positions to engage colleagues and students in a global social movement for critical social work’. Here, I wondered if I had managed some resistance by retaining at least some of my intended meaning, or what Orwell (1949), in *1984*, coined as ‘thoughtcrime’ (p. 10). Thoughtcrime, according to Orwell, is a concept that must be circumvented at all costs. It not only prevents challenging the ideas or operations of The Party [i.e., the ruling class university managers], but outlaws even thinking about challenging. As one of the characters involved in refining the new language explains: … ‘in the end we shall make thoughtcrime literally impossible, because there will be no words in which to express it’ (Orwell, 1949, p. 29). This is perhaps why Davies and Bansel (2010) emphasise the vital role of academics to ‘speak into existence’ alternative discourses, understandings and ways of doing and being (p. 5).

Interestingly, in summing up, the panel noted that ‘in general, discussion of [my] leadership in university contexts appears to lack strategic connectivity to wider School, Faculty, and institutional agendas and practices i.e., leadership and lobbying in multiple directions’. So, while my ‘strategic leadership’, to advance a critical social work agenda was clearly and consistently articulated in my application, yet constantly problematised or denied by reviewers, at the end of the day, ‘strategic leadership’ according to them requires applicants to simply trot out reference to the senior executives’ [i.e., the Party’s] policies and strategies [i.e., Newspeak]. Hence true ‘leadership’ in this scheme seems to involve strategically *following* more institutionally (hierarchically) significant others. By way of revision, a few brief references were made to my university’s Newspeak, while all other examples of my leadership and lobbying that were consistent with an emancipatory change agenda for change, were ‘strategically’ removed, which seemed to satisfy the panel in their final deliberations.

## A framework for analysing the neoliberal managerial obliteration of academic practice

The elimination of academic work and scholarly outputs when applying for recognition of academic achievement is symptomatic of a marketised university system that has become profoundly out of balance (Ablett et al., 2022; Davis, 2011; Fraser & Taylor, 2016; Hil, 2012; Li et al., 2016; Macias, 2015; Morley et al., 2017; Robinson & Macfarlane, 2021; Sims, 2020; Smyth, 2017; Todd et al., 2017). There are countless ways that neoliberal managerialism seeks to undermine, displace, disrupt, dismantle, and quash academic work (especially teaching and research), in universities, which effectively ‘chok[es] the very life out of… academic[s] (Hil, 2012, p.105). The attacks on academic values and practices have intensified within Australian universities since the COVID-19 pandemic, with the sudden closure of national borders that effectively derailed the revenue from international student markets. Universities had already become increasingly reliant on international student markets, due to being systematically underfunded by successive governments over several decades (Welsch, 2022). Despite economic modelling pointing to a $19 billion revenue deficit in the higher education sector over a three-year period from 2020, the former Morrison Government excluded public universities from JobKeeper financial support. The result was the further decimation of the sector, with a loss of 40,000 (or 1 in 5) jobs from the tertiary education sector in Australia during that period (Napier-Ramans & Wilkins, 2021).

The narrative ethnography presented in this paper is but one microcosmic example that exemplifies a process that Arendt (1963) would have described as ‘the banality of evil’ (p. 118). While neoliberal managerialism and its effects are palpable and overwhelming in many ways (such as sustained cuts and exclusion from Government financial support during a time of crisis), other impacts of neoliberal managerialism, such as those evident in my account, are more subtle and sinister. It matters that the mentors and reviewers of this scheme do not do academic work, and therefore, do not understand or value it. It matters that they eschew evidence of impact of academic work, instead valorising the technical aspects of management. It matters because they don’t recognise the processes of colonisation and harm that they are perpetrating, and so dutifully carry out as if an act of benevolence. They could not, for example, differentiate between the books, because they don’t produce scholarship. They did not see the feedback from graduates as speaking directly to the fundamental purpose and importance of education, because they don’t teach and have never shaped graduates’ practice. This paper is not a comment about these individuals, or senior executives more broadly, who are now part of the colonising machine within universities, but about a system that is wholly implicated in neoliberal managerialism and rapidly becoming dysfunctional as a result (Hil et al., 2022; Sims, 2020).

It is in the mundane, everyday occurrences that the ‘banality of evil’ operates (Arendt, 1963, p. 118). Arendt (1963) used this phrase to describe how she viewed history’s most extreme violence as being perpetrated by ordinary people who simply failed to question the existing status quo; everyday people, rather than psychopaths or horrifying figures. For Bourdieu (2001), it’s this ‘malignant form of unconsciousness’ (Springer, 2012, p. 140) that is so destructive. Bourdieu saw the ‘processes of domination’ as ‘insidious and difficult to identify; often leaving those who are disadvantaged by the system to ascribe their problems to themselves’ as constituting a form of ‘symbolic violence’ (Brough et al., 2020, p. 515). In this case, the most significant achievements from an academic career spanning more than two decades were erased and replaced with a set of generic management techniques that can be googled, appropriated and described by any manager, regardless of academic expertise, achievement, or impact. The managerial mantra that a good manager can manage anything falls short with the awkward gaps and deficiencies in fundamental academic knowledge, skills, experience, and scholarly achievements, exhibited by many senior executives who hold powerful roles within our academic institutions in increasing numbers. Indeed, there are now at least two Vice Chancellors in Australia that have a purely administrative track record and many more Pro-Vice Chancellors and other corporate/senior executives within Australian universities who lack academic experience and expertise (Croucher & Woelert, 2021). Indeed, given the administrative revolution (Croucher & Woelert, 2021; Ablett et al., 2022), perhaps in order for senior executives to justify their roles in universities, it is necessary for them to redefine what constitutes academic leadership (and by extension, the very nature of academia and its purpose) by obscuring the value and purpose of academic work. However, this is the kind of ‘social amnesia that erases critical thought, historical analysis and any understanding of broader systemic relations’ (Giroux, 2014, p. 2).

Habermas’ (1984) concepts of the ‘lifeworld’ and ‘system’ offer a useful framework to understand the dire implications of this situation for higher education and society (p. 35). Habermas (1984) argued that every society has at least two domains: one that is systemic, which operates according to a strategic rationality (i.e., markets, bureaucracy, rationality (i.e., instrumental rationality)). The other domain or logic is that of the lifeworld (i.e., lived experience), which depends on shared values and communication, and non-strategic, communicative discourse and ethics. In contemporary (neoliberal) capitalist contexts, these two modes of logic have become increasingly separated, causing significant problems (Baianstovu & Ablett, 2020).

While the system and lifeworld can co-exist, in the university context, if systemic logic undermines the legitimate terrain of the lifeworld in facilitating ethical learning, discourses, and outcomes, the result will be a higher education system that produces students, academics, graduates, practitioners, and citizens who only think in strategic terms (i.e., neoliberal subjects). As Baianstovu and Ablett (2020) explain, ‘an increasing breech between the lifeworld and system, whereby the system contributes to the enforced decomposition of the lifeworld… [results in] divisions… antagonism and disintegration …[of] democratic norms’ (pp. 456–457). Habermas would have referred to this situation as a misdirected systemic colonisation of the educational lifeworld. As Baianstovu and Ablett (2020) suggest:

The incursion can be can be seen in… the reduction of educational quality to performance metrics and market demand; an increase in standardised testing at the expense of imparting critical analysis and dialogical learning; a focus on techniques at the expense of engaging and reflectively conversing with people; the increasing mediation of technology in teaching at the expense of face-to-face interaction; and the reduced autonomy of social work educators in having to comply with managerial systems of reporting and audit that leave little time for creative curricular developments. (p.456)

## Conclusions

Ultimately, the purpose of education, particularly higher education, is to contribute to an ethically driven democratic society (Giroux, 2011). Brookfield (1995) relatedly proclaims: ‘we teach to change the world’ (p. 1). Without radical reform, or indeed a revolution in higher education (Ablett et al., 2022), this reflection on my academic experience and practices highlights that the uncoupling of the ethical and substantive dimensions and imperatives of the (educational) lifeworld, from systemic (neoliberal managerial) strategising, results in an Orwellian nightmare. When the system colonises the educational lifeworld, it intrudes, impedes, and distorts higher education, causing it to go into a state of paralysis. The current state of higher education, following more than a decade of hostile neoliberal policy from the former Coalition Government, is a case in point.

It is hoped that the recent election of the new Labor government will provide some ways forward for higher education, with an additional 20 000 Commonwealth Government Supported places recently announced for students from socio-economically disadvantaged backgrounds (including students from remote areas, those who are first in the family to study at university, First Nations students, and students with a disability) (Australian Government Department of Education, 2022). It is also hoped that one of the contributions of this article—to unmask the Orwellian technologies (Orwell, 1949), the symbolic violence (Bordieu, 1990), and the banality of evil (Arendt, 1963) operating within contemporary Australian universities—augments academics, who grapple with the paralysing effects of neoliberal managerialism with a critical analytical framework that recognises, critiques, and resists similar colonising operations in their own contexts. Critical analysis of these technologies of injustice gives rise to developing alternatives, counterhegemonic discourses, and the capacity for academics to resist and transgress.

## References

[CR1] Ablett P, Morley C, Fraser H, Kamali M, Jönnson J (2022). Progressive revolutionary change and the democratic university: Reimagining higher education. Revolutionary social work.

[CR2] Abramovitz M, Zelnick J, Franklin C (2021). Neoliberal managerialism and the human services. Encyclopedia of social work.

[CR3] Arendt H (1963). Eichmann in Jerusalem: A report on the banality of evil.

[CR4] Australian Government Department of Education (2022). *20,000 additional Commonwealth supported places.* Retrieved from https://www.education.gov.au/higher-education-funding/commonwealth-grant-scheme-cgs/20000-additional-commonwealth-supported-places

[CR5] Baianstovu R, Ablett P, Morley C, Ablett P, Noble C, Cowden S (2020). The transformation and integration of society: Developing social work pedagogy in social work education. The Routledge handbook of critical pedagogies for social work.

[CR6] Bentley, N. (2017, November 22). What does ‘Orwellian’ mean, anyway? *The Conversation*. Retrieved from https://theconversation.com/what-does-orwellian-mean-anyway-87404

[CR7] Berg L, Huijbens E, Larsen G (2016). Producing anxiety in the neoliberal university. The Canadian Geographer.

[CR8] Blackmore J (2014). Cultural and gender politics in Australian education, the rise of edu-capitalism and the ‘fragile project’ of critical educational research. Australian Educational Researcher.

[CR9] Bourdieu P (2001). Masculine domination.

[CR10] Brookfield S (1995). Becoming a critically reflective teacher.

[CR11] Brookfield S (2017). Becoming a critically reflective teacher.

[CR12] Brough M, Kippax R, Adkins B, Morley C, Ablett P, Noble C, Cowden S (2020). Navigating the politics and practice of social work research with advice from Pierre Bourdieu. The Routledge handbook of critical pedagogies for social work.

[CR52] Bordieu P (1990). The Logic of Practice.

[CR13] Carter DM (2017). How and why I became a principal fellow of the higher education academy. Council of University Classical Department Bulletin.

[CR14] Connell R (2013). The neoliberal cascade and education: An essay on the market agenda and its consequences. Critical Studies in Education.

[CR15] Croucher, G., & Woelert, P. (2021). Administrative transformation and managerial growth: A longitudinal analysis of changes in the non-academic workforce at Australian universities. *Higher Education*, 1–17.

[CR16] Davies B, Bansel P (2010). Governmentality and academic work: Shaping the hearts and minds of academic workers. Journal for Curriculum Theorizing.

[CR17] Davis D (2011). Constructing fear in academia: Neoliberal practices at a public college. Learning and Teaching.

[CR18] Ellis C, Adams TE, Bochner AP (2011). Autoethnography: An overview. Historical Social Research / Historische Sozialforschung.

[CR19] Fook J (2016). Social work: A critical approach to practice.

[CR20] Fraser H, Taylor N (2016). Neoliberalism, universities and the public intellectual: Species, gender and class and the production of knowledge.

[CR21] Freire P (1972). Pedagogy of the oppressed.

[CR22] Garrett PM (2010). Examining the ‘conservative revolution’: Neoliberalism and social work education. Social Work Education.

[CR23] Giroux H (2011). On critical pedagogy.

[CR24] Giroux H (2014). Neoliberalism’s war on higher education.

[CR25] Giroux H (2015). Dangerous thinking in the age of new authoritarianism.

[CR26] Habermas J (1984). The theory of communicative action.

[CR27] Hil R (2012). Whackademia: An insider’s account of the troubled university.

[CR28] Hil R, Lyons K, Thompsett F (2022). Transforming universities in the midst of global crisis.

[CR29] Howie M (2020). In ‘obey’-ance: One principal’s critically reflexive engagement with research in a culture of compliance. Australian Educational Researcher.

[CR30] Li N, Marsh V, Rienties B (2016). Modelling and managing learner satisfaction: Use of learner feedback to enhance blended and online learning experience. Decision Sciences Journal of Innovative Education.

[CR31] MacDonald-Vemic A, Portelli J (2020). Performance power: The impact of neoliberalism on social justice educators’ ways of speaking about their educational practice. Critical Studies in Education.

[CR32] Macias T (2015). Between a rock and a hard place: Negotiating the neoliberal regulation of social work practice and education. Alternative Routes.

[CR33] Morley C (2019). Social work education and activism. Routledge handbook of critical social work.

[CR34] Morley C, Ablett P, Morley C, Ablett P, Noble C, Cowden S (2020). Henry Giroux’s vision of critical pedagogy: Educating social work activists for a radical democrac*y?*. The Routledge handbook of critical pedagogies for social work.

[CR35] Morley C, Ablett P, Macfarlane S (2019). Engaging with social work: A critical introduction.

[CR36] Morley C, Ablett P, Noble C, Cowden S (2020). The Routledge handbook of critical pedagogies for social work.

[CR37] Morley C, Ablett P, Stenhouse K (2019). Technicist Education: Paving the way for the rise of the social work robots?. Critical and Radical Social Work.

[CR38] Morley C, Macfarlane S, Ablett P (2014). Engaging with social work: A critical introduction.

[CR39] Morley C, Macfarlane S, Ablett P (2017). The neoliberal colonisation of social work education: A critical analysis and practices for resistance. Advances in Social Work and Welfare Education.

[CR40] Napier-Ramans, K., & Wilkins, G. (2021, October 28). How the pandemic reshaped universities—and delivered on a Coalition dream. *Crikey*. Retrieved from https://www.crikey.com.au/2021/10/28/university-covid-19-funding-cuts/

[CR41] Orwell, G. (1949). *Nineteen Eighty-Four: A Novel*. (Also published as 1984), Secker & Warburg, London.

[CR42] Robinson K, Macfarlane S (2021). ‘Resistant to change?’ Using critical reflection to analyse positionality within a neoliberal academic environment. Critical and Radical Social Work.

[CR43] Selkrig M, Manathunga C, Keamy R (2021). Research is…making the emotional dimensions of academics’ research visible. Australian Educational Researcher.

[CR51] Shahjahan R (2014). From 'no' to 'yes'" Postcolonial perspectives on resistance to higher education. Discourse: Studies in the Cultural Politics of Education.

[CR44] Simpson G (2020). Book review: The Routledge handbook of critical pedagogies for social work. Social Work Education.

[CR45] Sims M (2020). Bullshit towers: Neoliberalism and managerialism in universities in Australia. Peter Lang.

[CR46] Smyth J (2017). Toxic university.

[CR47] Springer S (2012). Neoliberalising violence: Of the exceptional and the exemplary in coalescing moments. Area.

[CR48] Todd S, Barnoff L, Moffatt K, Panitch M, Parada H, Strumm B (2017). A social work re-reading of students as consumers. Social Work Education.

[CR49] Weber M (1968). Economy and society: Outline of interpretive sociology.

[CR50] Welsch A (2022). COVID crisis, culture wars and Australian higher education. Higher Education Policy.

